# Effects of a Sports-Oriented Primary School on Students’ Physical Literacy and Cognitive Performance

**DOI:** 10.3390/jfmk3030037

**Published:** 2018-06-27

**Authors:** Yolanda Demetriou, Joachim Bachner, Anne K Reimers, Wiebke Göhner

**Affiliations:** 1 Department of Sport and Health Sciences, Technical University of Munich, 80992 Munich, Germany; 2 Department of Human Movement Science and Health, Chemnitz University of Technology, 09111 Chemnitz, Germany; 3 Katholische Hochschule Freiburg, 79104 Freiburg, Germany

**Keywords:** physical activity, health, students

## Abstract

As only a small group of children fulfil the guidelines for physical activity, interventions are necessary to promote active lifestyles. We examined the effects of a sports-oriented primary school (*N* = 79) in comparison to a regular primary school (*N* = 90) on students’ physical literacy and cognitive performance. To evaluate the implementation of the sports-oriented school curriculum a process evaluation was conducted, in which the school curriculum was analysed and guideline-based interviews were carried out with the schoolteachers and the school director. To measure students’ physical literacy and cognitive performance several tests were used. Small positive effects of the sports-oriented primary school on students’ physical literacy were shown in standing long jump and attitudes towards physical activity. There were no differences between the groups regarding cognitive performance. This study provides the first insights on how a sports-oriented school can promote students’ physical literacy in the future. The results are in line with previous research that shows that when children spend more time in physical education and overall physical activities at school, no negative consequences result for their cognitive performance. In future, long-term evaluations of the effects of sports-oriented schools are required to receive valid results on the effects on students.

## 1. Introduction

In modern societies, chronic diseases represent the most substantial problem in the health system [1]. Systematic reviews show that regular physical activity is associated with positive health effects in children and adolescents [2]. In order to achieve these benefits, children require a minimum amount of regular physical activity of at least 60 min per day [3]. As only a small group of children fulfil the recommended guidelines for physical activity [4,5], interventions are necessary to promote active lifestyles starting from a young age [6].

There are several reasons why schools offer the ideal setting to implement interventions promoting physical activity. First, the central task of school is to improve the cognitive and academic performance of students and at the same time to provide them with the skills and abilities to lead a healthy lifestyle [7]. Second, physical education takes over a major part in the education of the physical through methods to increase physical fitness [8] while at the same time it educates the students through the physical, in terms of physical activities that provide learning, socialization opportunities, and affective outcomes [9,10]. Third, children and adolescents who lead an inactive lifestyle can be exposed to the intervention programmes. Fourth, since students spend a large amount of time at school, in addition to physical education lessons there are widespread opportunities to promote physical activity such as during recess or extracurricular physical activity programmes. 

Physical literacy captures the essence of the basic skills children and adolescents should attain in order to be physically active and participate in sports [11,12]. Several models have been developed that try to describe the construct of physical literacy. Whitehead [13] defines physical literacy as “the motivation, confidence, physical competence, knowledge and understanding to maintain physical activity throughout the lifecourse”. Based on Lloyd, Colley and Tremblay [11] physical literacy represents the successful interaction of four inter-related core domains: (a) physical fitness (cardiovascular fitness, muscular strength and endurance, flexibility, and coordination); (b) fundamental motor skills (e.g., catching and throwing a ball); (c) physical activity behaviours, and (d) psycho-social/cognitive factors (attitudes, knowledge, and feelings). Numerous studies have shown the importance of these variables for children’s and adolescents’ health [2]. Overall, physical literacy is a basic requirement to receive lifelong health benefits by being physically active and participating in sports. Against the background of the concept of physical literacy, it becomes clear that the transportation of these core domains is one essential task of school and physical education in order to promote an active and healthy lifestyle.

The question rises whether the promotion of physical literacy comes at the expense of students’ cognitive and academic performance. A systematic review suggests that acute bouts of physical activity facilitate children’s performance on tests that measure attention, memory, rapid decision making, and planning [14]. Additionally, recent well-designed experiments provide evidence that chronic exercise interventions benefit specific aspects of children’s mental functioning [14]. Finally, it can be assumed that the executive skills children acquire during physical education may transfer to academic tasks [15].

In order to promote students’ physical literacy, a large number of intervention programmes have been carried out in the school setting [16]. Nevertheless, the effects of these programmes have been small for the most part. Based on these results, it can be assumed that the implementation of temporary intervention programmes is not enough to achieve the abovementioned goals. Rather, intensive programmes spread over the entire school day are promising. Therefore, in this pilot study, we examine the effects of a sports-oriented primary school with a physical activity intensified curriculum and a physical activity-friendly school environment on students’ physical literacy and cognitive performance. In a first step, we carried out a process evaluation to develop a better understanding of the implemented curriculum of the sports-oriented school. In a second step, we analysed whether relative to the students of a control school, the students of the sports-oriented primary school would exhibit significantly higher values in the four core domains of physical literacy: (a) physical fitness; (b) fundamental motor skills; (c) physical activity behaviours; and (d) psycho-social/cognitive factors. Additionally, we assumed that the promotion of physical literacy would leave the students’ cognition unimpaired or would even enhance it. Accordingly, we predicted that there would be no significant differences in cognitive performance between the students of the two schools.

## 2. Materials and Methods

### 2.1. Participants

Participants were recruited from a sports-oriented primary school (intervention group, IG) and a regular primary school without a sports-orientation (control group, CG) in the curriculum. The sports-oriented school is the only primary school in south Germany that provides daily physical education lessons and overall physical activity bouts over the entire school day. To examine the effects of this school type on students’ physical literacy and cognitive performance, all students enrolled in the sports-oriented were included in the study. As a comparison, a primary school also located in the south of Germany, in a comparable city regarding size and population, was chosen. Nevertheless, because the control school was larger, a random selection of four out of ten classes existing were included in the study. The study sample consisted of *n* = 169 primary school students (sports-oriented school: *n* = 79; control school: *n* = 90) with a mean age of 8.06 years (SD = 1.21), at the time of the first measurement (see Table 1). Overall, more boys (*n* = 101; 59.8%) than girls participated in the study. One first, one second, one third and one fourth class from each school were included.

The sports-oriented primary school is the first private sports-oriented school in Germany. It follows the state curriculum and has been recognised by the German state. The school curriculum comprises daily 90-min physical education lessons active recess opportunities and additionally, the non-sport subjects are taught in an active way. Students in the regular primary school receive three 45-min sessions of physical education. No specific efforts to increase students’ physical activity levels during school hours are made.

### 2.2. Instruments

#### 2.2.1. Process Measures

To evaluate the implementation of the sports-oriented school curriculum a process evaluation was conducted. In the first step, the curriculum of the school was analysed. Additionally, guideline-based interviews were carried out with the school teachers and the school director to gain information about the frequency, duration and content of the physical education lessons, recess and the physical activity during regular classes.

#### 2.2.2. Outcome Measures

The following tests were used to measure the children’s values in the four domains of physical literacy and cognitive performance. Because children in the first two grades of school are not yet able to read and write sufficiently, questionnaire items were read to the students and they were asked to give their answer.

To assess physical fitness several tests of the German motor performance test *DMT 6–18* [17,18] were used. This instrument was developed within the scope of the German Society of Sport Science. Altogether, it consists of eight tests that measure students’ endurance, strength, speed, coordination and flexibility. For this study, standing long jump (strength; cm jumped), sit-ups (strength; number completed in 40 s), the 6-min-run (endurance; metres run in 6 min), stand-and-reach flexibility (flexibility; reached cm over or under the toes) and backwards balancing on bars with different widths (coordination; 0–8 points) were chosen. In this study, the test-retest reliability of the applied tests over approximately seven months ranged between r_tt_ = 0.64 and r_tt_ = 0.83.

Student’s fundamental motor skills were measured using several tests of the basic motor competences test (*MOBAK-1*) for children in grades one and two [19] and basic motor competences test (*MOBAK-3*) for children in grades three and four [20] test batteries. Each of the batteries contains eight tests differentiating between assessing the ability to move an object or to move oneself. We concentrated on testing skills in moving an object and used the tests throwing, catching, dribbling and bouncing for the children in the first two grades. The older children performed the same tests, only throwing and catching was assessed within one test. The test-retest reliability ranged between r_tt_ = −0.06 and r_tt_ = 0.35.

Physical activity behaviour was measured with the question “On how many days of last week were you physically active for more than 60 min?” [21,22]. Test-retest reliability was r_tt_ = 0.28. Standardised questionnaires were used to measure the psycho-social factors of physical literacy. Students’ motivation towards physical education was assessed with a questionnaire based on the *Intrinsic Motivation Inventory* [23]. Their attitudes towards physical education was assessed with the *Attitudes towards physical education* (ESU) questionnaire [24] whereas health-related fitness knowledge was measured with a previously developed questionnaire [25]. Lower values represent higher motivation and attitude. Knowledge is described by the percentage of correct answers. For the motivation scale the internal consistency measured by Cronbach’s alpha was 0.62. Internal consistencies for attitudes towards physical education and fitness knowledge were 0.57 and 0.64, respectively.

Cognitive performance was assessed with two computer-based tests: (a) the Simon task in the form of a Dots test [26], which again contains three subtests; and (b) the Flanker test [27]. In a Simon task, a single item is presented in one of two locations (to the left or right of fixation). The participant must indicate the identity of the item with a left or right keypress. Because of the natural tendency to respond in the direction of task-relevant stimuli, response time is faster when the required response (e.g., right keypress) is congruent with the location of the stimulus (e.g., right side of fixation). The Flanker task is widely used to measure interference control. This task calls for the participant to identify a target item defined by its location while ignoring one or more distracting items that flank the target and whose identities may activate the correct (on congruent trials) or incorrect (on incongruent trials) response. For the Dots tests and the Flanker test the inverse efficiency score [28] was calculated, which accounts for speed and accuracy of the students’ answers. Additionally, a reaction test was carried out to make sure that differences in performance between intervention group (IG) and control group (CG) are not only based on a difference in reaction speed. Test-retest reliability for the Dots test was by average at 0.69. The respective value for the Flanker test was 0.11 and for the reaction test 0.41. 

### 2.3. Procedure

During the academic year 2014/2015, a pilot study was conducted to examine the effects of a sports-oriented primary school on students’ physical literacy and cognitive performance in comparison with students of a regular primary school. In order to receive more reliable results, data were assessed twice. Measurements in both IG and CG took place between December 2014 and July 2015. For the process evaluation of the curriculum of the sports-oriented primary school, teachers and the school director were interviewed.

Information about the study and request for participation were sent to the school directors by the regional council. CG teachers were informed that they were participating in a study examining the development of students’ motor and cognitive performance from grade one to four. Students’ parents were informed about the study and provided their consent for their children to participate in the programme. All procedures performed in this study were in accordance with the 1964 Declaration of Helsinki and its later amendments. The ethics department of the medical faculty at the University of Tübingen in Germany, the regional council, school directors and teachers approved the implementation of this study (Approval code: 155/16 S; approval date: 12 May 2014). 

### 2.4. Data Analysis

Analyses were performed using SPSS version 23 (IBM, Armonk, NY, USA). Missing values in the scales assessing the psycho-social factors of physical literacy were replaced by the expectation maximization technique. After checking for normal distribution and potential multicollinearity of the variables, multivariate analyses of variance (MANOVA, Sha Tin, HongKong) were conducted to test for differences between the students of the two schools on the examined constructs at the respective time points. The guideline-based interviews were analysed by summarising the teachers’ and the director’s answers regarding the frequency, duration and content of the physical education lessons, recess and the physical activity during regular classes. Additionally, reasons for not being able to comply with the physical activity aims of the school were listed.

## 3. Results

### 3.1. Process Evaluation

Based on the written curriculum provided by the school director, it became clear that the vision of the sports-oriented primary school is to provide students with the opportunities and time to achieve the necessary amounts of daily physical activity and movement skills in order to counter against the health consequences of a sedentary lifestyle. Additionally, the school aims to promote students’ cognitive performance through regular physical activity intervals and therefore eventually enhance the students’ academic performance. 

The structured interviews with the school teachers provided an insight of the elements of the physical activity intensified curriculum and the vision of the school. The broad corridors in the school building provided indoor opportunities for games and physical activities especially on rainy days. On days with good weather, the students spent their time during recess mostly on the schoolyard. Even though the schoolyard provides enough space for movement, the school teachers requested several improvements. For example, they observed that some of the students did not know what to do during recess and therefore did not engage in physically active behaviours. More traditional playground elements such as swings or a slide and a climbing opportunity would be further possibilities to promote physical activity in less active children. Additionally, the organisation of the existing play equipment (balls, skipping ropes) needed further improvement in a way that the students can independently have access to it at any time during the school day. 

Eight 45-min physical education lessons were provided in each class over a week including two hours of swimming lessons every Friday. During physical education the teachers set a focus on the promotion of coordinative skills in the first two grades using basic elements such as balancing and swinging elements but also modern elements such as wave boards and roller-skates. In grade three and four, several classical sports were taught such as track and field or soccer. 

An important aspect of the sports-oriented school was that the classroom teachers integrated movement elements during regular class hours so that physical activity was not only restricted to physical education and recess. Every morning the school begins with a movement element. For example, one classroom teacher developed a “health box” in which several fitness exercises such as sit-ups, jumping jack, or jump and reach are described. Every morning the children chose five to six exercises that are carried out at the beginning of the first school hour. Another example could be found in lessons teaching foreign languages. While introducing new adjectives, the teachers named several words. Each time the students recognised an adjective they had to stand on their desk. Finally, sedentary behaviour was reduced in class because children were allowed to stand up from their seat whenever they wanted to have a glass of water or to go to the toilet. 

The final part of the interview with the teachers addressed the motivation of the students to participate at physical activities during the entire school day. Overall, the teachers emphasised that the majority of the children were always happy to be active. Nonetheless, when a student expressed that he or she was tired, the student was not obliged to participate as long as he or she did not disturb the rest of the class. Few exceptions also existed where some children were never happy to engage in physical activities irrespective of the time of day or whether it was an activity during regular class or physical education. 

### 3.2. Outcome Measures 

The comparison analysis between the sports-oriented and the regular primary school is shown in Table 2 and Table 3. Concerning the students’ physical literacy, small positive effects of the sports-oriented school in comparison to the regular primary school were observed. 

Regarding the first core domain of physical literacy, physical fitness, students of the sport-school exhibited significantly higher values in standing long jump at both time points. Group membership explained 7.3% and 4.9% of the variance found in this test at the respective time points. Additionally, there were differences in balancing backwards and 6-min run in favour of the sports-oriented school at both time points, although not reaching statistical significance. For each applied test, the values of our sample were in the range of the reference sample [18].

Regarding psycho-social factors, the students of the sports-oriented primary school showed significantly better values in their attitude towards physical education in comparison to the regular primary school at both time points. Group membership was responsible for 6% and 10.1% of the variance at the respective time points. No significant differences were found for health-related fitness knowledge and their motivation towards physical education between both schools.

The students’ values regarding fundamental motor skills and physical activity behaviours could not be assessed reliably. Since acceptable reliabilities build the basis for every scientific examination, it was refrained from analysing the students’ performance in the MOBAK tests and their physical activity levels. 

Concerning the cognitive performance, no significant between-group differences were found. None of the dots tests discriminated between the groups. Significant differences in reaction speed could neither be found. The values in the Flanker test were not analysed, because test-retest reliability was not satisfying. 

## 4. Discussion

This study is the first in Germany that reported the effects of a sports-oriented primary school on students’ physical literacy and cognitive performance and that evaluated the implementation of a sports-oriented primary school curriculum. 

The interviews revealed the ambition of the school director and the school teachers to contribute to the achievement of the required daily physical activity levels in the students to obtain positive health outcomes, to promote students’ cognitive and academic performances and to improve the students’ movement skills. Unfortunately, the effects of the sports-oriented curriculum on physical activity levels could not be analysed within this study, because the reliability of the measure on physical activity was not satisfactory. The interviews further revealed that, from the teachers’ point of view, for the successful implementation of a sports-oriented school curriculum that promotes daily physical activity, more than just some additional physical education lessons is needed. Teachers assumed that to foster physical activity during the whole school day encouragement and motivation of students are requisite. The influence of motivational support on physical activity during school days was also demonstrated in interventional studies [29,30].

Furthermore, activity-friendly built environmental structures are needed that facilitate physical activities during the school day in an indoor and outdoor environment, respectively. This is in line with findings from other empirical studies with quantitative designs [31,32] showing that a larger number of outdoor facilities like soccer fields and loose play equipment like skipping ropes at school was associated with higher odds of being physically active in boys and girls.

In summary, to establish an activity-promoting school concept besides a sports-oriented curriculum and supportive social environments (teachers willing to support physical activity during the whole school day) activity-friendly built environmental structures providing space and equipment for physical activities are relevant. 

Regarding the quantitative study results, small positive effects in physical literacy can be attributed to the sports-oriented school. In comparison to the control school, the students of the sports-oriented school had significantly higher levels in standing long jump and a more positive attitude towards physical activity at both measurement time points. Furthermore, positive tendencies were observed in the sports-oriented school in comparison to the control school in the 6-min run, balancing backwards and sit-ups (only in T1). Finally, there were no significant between-group differences in motivation towards physical education, fitness knowledge or cognitive performance. Slightly better values of one of the groups were always dependent on subtest and measurement time point. Thus, no indications towards a potentially consistent pattern could be found for these aspects.

The effects of the sports-oriented school on students’ physical literacy were small. Based on the information received in the interviews with the school teachers, this could be due to several reasons. It is likely that children from the control school compensated their sedentary behaviour to a great extent in the afternoon hours, whereas students of the sports-oriented school compensated their additional amount of physical activity during school time with a reduction of physical activity during the after-school hours. This is in accordance with the “Activitystat” hypothesis suggesting that the individual’s amount of physical activity and energy expenditure is controlled over time to remain constant. Therefore, an individual’s increase in physical activity in one setting is accompanied by a compensatory reduction of physical activity in another setting to maintain overall physical activity level [33,34]. Similarly, in a study in Denmark comparing physical activity levels of 1st to 6th grade students from sport and normal schools, no differences in the overall physical activity levels were found. The overall physical activity levels remained stable as the students compensated the additional physical activity during school in the after school hours [35]. Nevertheless, these are only assumptions that need to be systematically examined in future studies.

In our study, there were no negative effects of additional physical education lessons on students’ cognitive performance. This is in line with previous studies [35,36], in which no differences existed between the students of the sports-oriented and the regular school in their cognitive performance. Also, in the study by Sallis et al. [37], in which the effects of a two year health-related school physical education programme on standardised academic achievement with 759 children was examined, it was shown that increasing physical education from 32 to 98 or 109 min/week did not reduce academic performance. Despite devoting twice as many minutes per week to physical education as controls, the health-related physical education programme did not interfere with academic achievement. In a recent meta-analytical study it was even shown that curricular physical education programmes even had a positive impact on cognitive functions [38]. Thus, rising concerns that increasing weekly time of physical education could lead to declines in academic performance due to less time remaining for other school subjects were not supported by our or other empirical studies. 

### Limitations

To the best of our knowledge, in Germany only one school exists that carries out a sports-oriented curriculum including daily physical education and active recess. Therefore, this pilot study is based on a small sample size and lacks the necessary power in the statistical analysis in order to provide solid findings. Nevertheless, this study provides a first glance into the possible effects of a sports-oriented school and gives first indications on positive effects on physical literacy. A longitudinal approach is needed to provide future directions to the development of whole-day sports-oriented schools. 

We used generic measurement instruments (*DMT 6–18, MOBAK*) to assess students’ physical fitness and motor abilities. *DMT 6–18* revealed high reliability warranting reliable conclusions on physical fitness. In contrast, *MOBAK* showed weak reliability values, and therefore no further conclusions were drawn based on these tests. Physical activity behaviour and psychological determinants were measured with questionnaires. Retest-reliability results of the physical activity behaviour question was very low. A timeframe of five months is very long to receive high reliability values. Nevertheless, values lower than r_tt_ = 0.40 do not allow conclusions to be drawn based on the results gained. Therefore, in future studies, besides larger study samples, objective measurements using accelerometers and systematic observation methods are mandatory to capture physical activity levels in primary school children. The reliability values of the tests measuring students’ cognitive functioning varied. While the dots test had satisfactory reliability, the flanker revealed very low values and therefore, the results based on this test were not further discussed in this paper. 

## 5. Conclusions

The effects of a sports-oriented primary school on students’ physical literacy could only be shown in the standing long jump and the attitudes towards physical activity. Nevertheless, the effects in these tests were small. In respect to cognitive performance, no significant differences between the two schools were observed. This is in line with previous research that showed that when children spent more time in physical education and overall physical activities at school, no negative consequences resulted on their cognitive performance [35,36,37]. In the future, long-term evaluations of the effects of sports-oriented schools are required to receive valid results on the effects on students. Nevertheless, this pilot study provides first insights on how to improve a sports-oriented school to promote students’ physical literacy in the future.

Physical inactivity levels are rising and schools need to adapt their curricula and environments in order to act against this. When also integrating improvements in activity-friendly environments, sports-oriented schools are a promising approach to contribute to higher levels of physical activity and less sedentary time in children during school hours, and to generally promote students’ physical literacy. Despite the greater time allocated to physical activity in the sports-oriented school (daily physical education, active recess), there were no negative consequences on students’ cognitive performance. 

The results have important implications for school health efforts. Even when it is not possible to allocate daily physical education in the school curriculum, several other low budget opportunities exist to transform the school into a more physical activity friendly environment (e.g., by provision of loose physical activity equipment during recess). The commitment of teachers and school directors is crucial when it comes to the implementation of such activities. It is important that the school directors encourage the school teachers to promote boosts of activities as a first action in the morning, as described by the school teachers of the sports-oriented school. Additionally, a culture of providing the children with more freedom and support to be physically active during regular class, by providing an activity-friendly school environment and loose play equipment, by avoiding sitting rules, and by combining learning with movement elements, seems warranted. 

## Figures and Tables

**Table 1:** Numbers of girls and boys participating at the study.

Class	Sports School		Control School	
	Girls	Boys	Girls	Boys
1. Class	*n* = 8	*n* = 15	*n* = 14	*n* = 12
2. Class	*n* = 6	*n* = 14	*n* = 12	*n* = 12
3. Class	*n* = 5	*n* = 16	*n* = 10	*n* = 11
4. Class	*n* = 7	*n* = 8	*n* = 6	*n* = 13
Total	*n* = 26	*n* = 53	*n* = 42	*n* = 48

**Table 2:** Means and standard deviations of the two schools at T1 and corresponding between-group results of the respective multivariate analyses of variance (MANOVAs).

Variable	Test	Sports School			Control School			MANOVA		
		Mean	SD	*n*	Mean	SD	*n*	F	Sig.	$\eta_{p}^{2}$
Physical fitness	stand-and-reach flexibility (cm)	0.91	6.93	75	−0.75	6.62	69	2.17	0.14	0.015
	Balance (points)	7.73	4.92	75	6.81	4.35	69	1.41	0.24	0.010
	Standing long jump (cm)	133.60	19.52	75	122.12	21.83	69	11.11	0.001 **^,a^	0.073
	Sit-ups (number)	16.16	6.90	75	15.49	4.65	69	<1	0.50	0.003
	6 min run (meters)	969.46	157.40	75	928.31	137.78	69	2.77	0.10	0.019
Psycho-social/cognitive factors	Knowledge (% correct)	67.83	20.92	69	71.73	18.89	81	1.44	0.23	0.010
	Attitude	1.87	0.39	69	2.10	0.49	81	9.48	0.002 **^,a^	0.060
	Motivation	1.61	0.53	69	1.60	0.48	81	<1	0.93	0.000
Cognitive performance	Dots 1	669.41	176.41	69	691.83	237.32	69	<1	0.53	0.003
	Dots 2	844.19	295.14	69	835.47	289.26	69	<1	0.86	0.000
	Dots 3	1424.74	493.90	69	1385.29	896.14	69	<1	0.75	0.001
	Reaction	495.82	128.40	69	461.62	113.44	69	2.75	0.10	0.020

Note: SD = standard deviation; ** *p* < 0.01, ^a^ = in favour of the sport school.

**Table 3:** Means and standard deviations of the two schools at T2 and corresponding between-group results of the respective MANOVAs.

Variable	Test	Sports School			Control School			MANOVA		
		Mean	SD	*n*	Mean	SD	*n*	F	Sig.	$\eta_{p}^{2}$
Physical fitness	stand-and-reach flexibility (cm)	−0.96	7.07	69	0.50	6.96	80	1.67	0.20	0.011
	Balance (points)	7.46	4.35	69	6.19	3.94	80	3.63	0.06	0.024
	Standing long jump (cm)	133.07	21.92	69	123.91	18.68	80	7.59	0.007 **^,a^	0.049
	Sit-ups (number)	19.28	6.50	69	19.31	5.63	80	<1	0.97	0.000
	6 min run (meters)	1003.01	122.46	69	991.50	133.40	80	<1	0.59	0.002
Psycho-social/cognitive factors	Knowledge (% correct)	78.47	21.07	72	76.05	18.49	86	<1	0.44	0.004
	Attitude	1.68	0.43	72	1.97	0.45	86	17.55	0.000 ***^,a^	0.101
	Motivation	1.69	0.56	72	1.56	0.54	86	1.94	0.17	0.012
Cognitive performance	Dots 1	640.37	178.17	71	699.68	658.17	78	<1	0.46	0.004
	Dots 2	759.62	254.53	71	735.36	212.00	78	<1	0.53	0.003
	Dots 3	1133.92	349.28	71	1090.64	470.31	78	<1	0.53	0.003
	Reaction	451.12	99.42	71	439.69	87.47	78	<1	0.46	0.004

Note: SD = standard deviation; ** *p* < 0.01, *** *p* < 0.001, ^a^ = in favour of the sport school.
